# Variability in the indication of brain CT scan after mild traumatic brain injury. A transnational survey

**DOI:** 10.1007/s00068-022-01902-5

**Published:** 2022-02-18

**Authors:** Alfonso Lagares, Ana María Castaño-Leon, Marion Richard, Parmenion Philip Tsitsopoulos, Julian Morales, Podaru Mihai, Vladislav Pavlov, Odile Mejan, Javier de la Cruz, Jean François Payen, BRAINI investigators, Maxime Maignan, Laurent Jacquin, Marion Douplat, Said Laribi, Philippe Pes, Patrick Ray, Jérémy Guenezan, Mustapha Sebbane, Frédéric Balen, Guillaume Durand, Cordelia Abric, María Teresa Lorca, Mariana Garcia Ponce, Maite Cuesta, Jose A. F. Alén

**Affiliations:** 1 Department of Neurosurgery, Hospital Universitario 12 de Octubre, Universidad Complutense de Madrid, Instituto de Investigación imas12, Madrid, Spain; 2 Department of Surgery, Facultad de Medicina, Universidad Complutense de Madrid, Madrid, Spain; 3 Department of Anesthesia and Intensive Care, University Grenoble Alpes, Centre Hospitalier Universitaire Grenoble Alpes, Grenoble Institut Des Neurosicences, INSERM, U1216 Grenoble, France; 4 Department of Neurosurgery, Hippokration General Hospital, Aristotle University School of Medicine, Thessaloniki, Greece; 5 Servicio de Urgencias, Hospital Universitario Gregorio Marañón, Madrid, Spain; 6 Servicio de Urgencias, Hospital Universitario del Tajo, Aranjuez, Spain; 7 bioMérieux, Medical Affairs, Chemin de LÓrme, Marcy-L´Étoile, France; 8 bioMérieux, Clinical Unit, Chemin de lÓrme, Marcy l´Étoile, France; 9 Instituto de Investigación imas12, Hospital Universitario 12 de Octubre, SAMID, Madrid, Spain

**Keywords:** Mild traumatic brain injury, Adult, Computed tomography, Clinical guidelines, Survey

## Abstract

**Purpose:**

Clinical guidelines have been developed to standardize the management of mild traumatic brain injury (mTBI) in the emergency room, in particular the indication of brain CT scan and the use of blood biomarkers. The objective of this study was to determine the degree of adherence to guidelines in the management of these patients across four countries of Southern Europe.

**Methods:**

An electronic survey including structural and general management of mTBI patients and six clinical vignettes was conducted. In-charge physicians from France, Spain, Greece and Portugal were contacted by telephone and email. Differences among countries were searched using an unconditional approach test on contingency tables.

**Results:**

One hundred and eighty eight physicians from 131 Hospitals (78 Spain, 36 France, 12 Greece and 5 Portugal) completed the questionnaire. There were differences regarding the in-charge specialist across these countries. There was variability in the use of guidelines and their adherence. Spain was the country with the least guideline adherence. There was a global agreement in ordering a brain CT for patients receiving anticoagulation or platelet inhibitors, and for patients with seizures, altered consciousness, neurological deficit, clinical signs of skull fracture or signs of facial fracture. Aging was not an indication for CT in French centres. Loss of consciousness and posttraumatic amnesia were considered as indications for CT more frequently in Spain than in France. These findings were in line with the data from the 6 clinical vignettes. The estimated use of CT reached around 50% of mTBI cases. The use of S100B is restricted to five French centres.

**Conclusions:**

There were large variations in the guideline adherence, especially in the situations considered to order brain CT after mTBI.

**Supplementary Information:**

The online version contains supplementary material available at 10.1007/s00068-022-01902-5.

## Introduction

Traumatic brain injury (TBI) is a common cause of admission to emergency departments. Most TBI patients admitted to EDs are classified as mild TBI (mTBI), defined by presenting with a Glasgow Coma Score (GCS) between 13 and 15 [1–3]. The main concern managing these patients is the ability to detect those who could deteriorate and therefore are in need of further assessment with a cranial CT [2, 4–6] and may eventually require an intervention. Several prediction rules and guidelines have been developed to estimate the risk of presenting an intracranial lesion, and thus be in risk of further deterioration. Some of these rules have gained international recognition, such as Canadian CT head rule [7], the New Orleans criteria [8], or the National Institute of Health and Care Excellence (NICE) guidelines [9]. These guidelines and others designed locally [10], use different clinical and examination factors to determine the need of performing cranial CT based on the risk of presenting intracranial lesions. These guidelines formulated indications for a cranial CT and therefore how management of mTBI should be carried out in a safe and effective manner.

Despite the development of clinical guidelines, different studies have reported wide variability in the management of mild TBI in developed countries and especially among European countries [11]. Though most patients could be managed without the need of a cranial CT, the variability in patient management determine CT overuse in these patients in many occasions.

Blood biomarkers could serve as objective tools to help in the assessment of the need for cranial CT in the management of these patients [3, 12]. Among a number of candidates S100B is the only biomarker included in a guideline, the Scandinavian guidelines [13, 14], to rule out the need for head CT in mTBI patients. However, the use of this guideline or the biomarker in southern European countries is unknown.

BRAINI is a prospective multicentre observational European Project aiming to determine the diagnostic accuracy of an automated assay for the measurement of serum GFAP and UCH-L1 in a cohort of patients with mTBI who received a CT scan as the standard of care [15]. As the goal of the BRAINI study is to assess the value of two novel biomarkers in the management of mTBI, we searched to assess how mTBI is currently managed in the emergency room in France and Spain, including Hospitals of different sizes, case load and variable resources. A sample of Hospitals from another two European countries (Greece and Portugal) was also obtained for comparison. Besides which situations should be considered for brain CT, we collected information from in-charge physicians regarding their management of mTBI patients in the emergency room.

The goal of this study was to investigate how mTBI is being managed in different Southern European countries with regards to which speciality is responsible for the management of mTBI patients, guideline use and adherence, indications for cranial CT and use of S100B in the management protocols in these countries.

## Methods

### Study design

A survey was designed using as a base the questionnaire developed by Center TBI investigators [11]. Certain modifications were made to include questions regarding structure of care of patients with mild TBI, including medical speciality in charge of care, guidelines used and estimated compliance, use of CT scan and S100B. Six clinical case vignettes were designed to cover different types of clinical situations and check guideline adherence as a separate part embedded in the questionnaire (See supplementary material).

Questionnaires were built into a REDCap database and sent in digital form. For Spain and France the questionnaire was translated into Spanish and French respectively. The English version was used for Portugal and Greece. Centres were contacted by phone personally or email to help get responses from them. National Spanish Society of Neurosurgery (SENEC) and French Society of Emergency Medicine contributed to the dissemination of these questionnaires.

Different members of departments at each hospital answered questionnaires to collect as much information as possible as well as different points of view from the same centre. Data from clinical vignettes were used as single answers from each clinician (one response per each physician filling this part of the questionnaire). For the rest of the questionnaire, answers from more than one physician per centre were averaged.

### Statistical analysis

For statistical analysis descriptive statistics were used. Categorical variables are presented as frequencies and percentages. Contingency tables were built to determine differences among countries regarding the conditions that prompted the ordering of a CT scan and the results from the clinical vignettes. A procedure designed to test significance in contingency tables using an unconditional approach (i.e. without applying first and omnibus test and further analysis of residuals) using a bootstrap correction for multiple testing as suggested by Garcia-Pérez et al. [16], was used for the analysis of differences among countries. All the analysis were performed using the computing environment R [R Core Team (2015), which is a language and environment for statistical computing. R Foundation for Statistical Computing, Vienna, Austria; http://www.R-project.org/]. Graphical analysis was performed by means of the *Likert* package [17] and analysis of contingency tables by means of *ACT* code for R [16].

## Results

### General results

One hundred and eighty eight (188) physicians from 131 Hospitals completed the questionnaire (Table 1). Spain (78 centres) and France (36 centres) had the highest frequency rates of answers. Physicians from different types of Hospitals were invited to answer the survey in both countries. In most occasions a neurosurgeon was available for consultation 24 h 7 days a week. Most Hospitals were public financed hospitals though in Spain and France also private financed hospitals were reached. In most centres there was a technician available on site to perform a cranial CT. In those not having this resource, a technician was available in less than an hour.

**Table 1:** Characteristics of 131 centers responding to the survey

	Spain (*n*, %)	France (*n*,%)	Greece (*n*, %)	Portugal	All
Hospital type					
Level 1 (few specialities, limited lab)	3 (4)	16 (45)	0	0	19 (15)
Level 2 (5–10 specialities)	22 (28)	17 (48)	2 (14)	1 (20)	42 (32)
Level 3 (all resources)	53 (68)	3 (7)	10 (86)	4 (60)	70 (54)
Public financing	74 (95)	31 (86)	12 (100)	5 (100)	122 (93)
Neurosurgeon available (24/7)	46 (59)	31 (86)	5 (42)	5 (100)	87 (66)
Trauma ICU on site	36 (46)	24 (67)	5 (42)	5 (100)	70 (53)
Technician to perform CT scan 24/7 on site	74 (95)	29 (81)	10 (83)	5 (100)	118 (90)
Who is responsible for the management of mild TBI					
Emergency medicine	81 (66)	38 (84)	2 (11)	2 (20)	125 (62)
Neurologist	2 (2)	0 (0)	0 (0)	0	3 (1)
Neurosurgery	25 (20)	3 (6)	11 (61)	3 (30)	43 (21)
Trauma surgeon	3 (2)	2 (4)	0 (0)	1 (10)	8 (4)
General surgeon	5 (4)	0 (0)	5 (28)	4 (40)	14 (7)
Intensivist	6 (4)	2 (4)	0 (0)	0	8 (4)
Observation area in the ER for mild TBI	74 (95)	34 (94)	9 (75)	4 (80)	121 (92)

More than one answer was allowed regarding especially responsible for mTBI management

There were differences between countries regarding the specialist in charge of mTBI patients. In some centres, more than one specialist was responsible for the management of mTBI patients in the emergency department setting, a factor that could increase local variability in the management. Emergency medicine is the main responsible speciality for the management of mTBI in the majority of French centres and most Spanish centres. In some Spanish centres Neurosurgeons are still responsible for directly managing mTBI, and there is a mix of several other specialities responsible in other Spanish centres. Portugal has a similar mix, as in the five centres that completed the questionnaire there is a mix of specialities responsible for the management of mTBI patients (general surgeons, neurosurgeons and emergency medicine physicians). In Greece mTBI seems to be managed mainly by neurosurgeons and general surgeons.

Most centres have an area in the ED for temporary observation of these patients. The median time for management of these patients in the ED of the different centres was 8 h (IQR = 3), without differences among the different countries surveyed.

### Adherence to guidelines

**Fig. 1:**
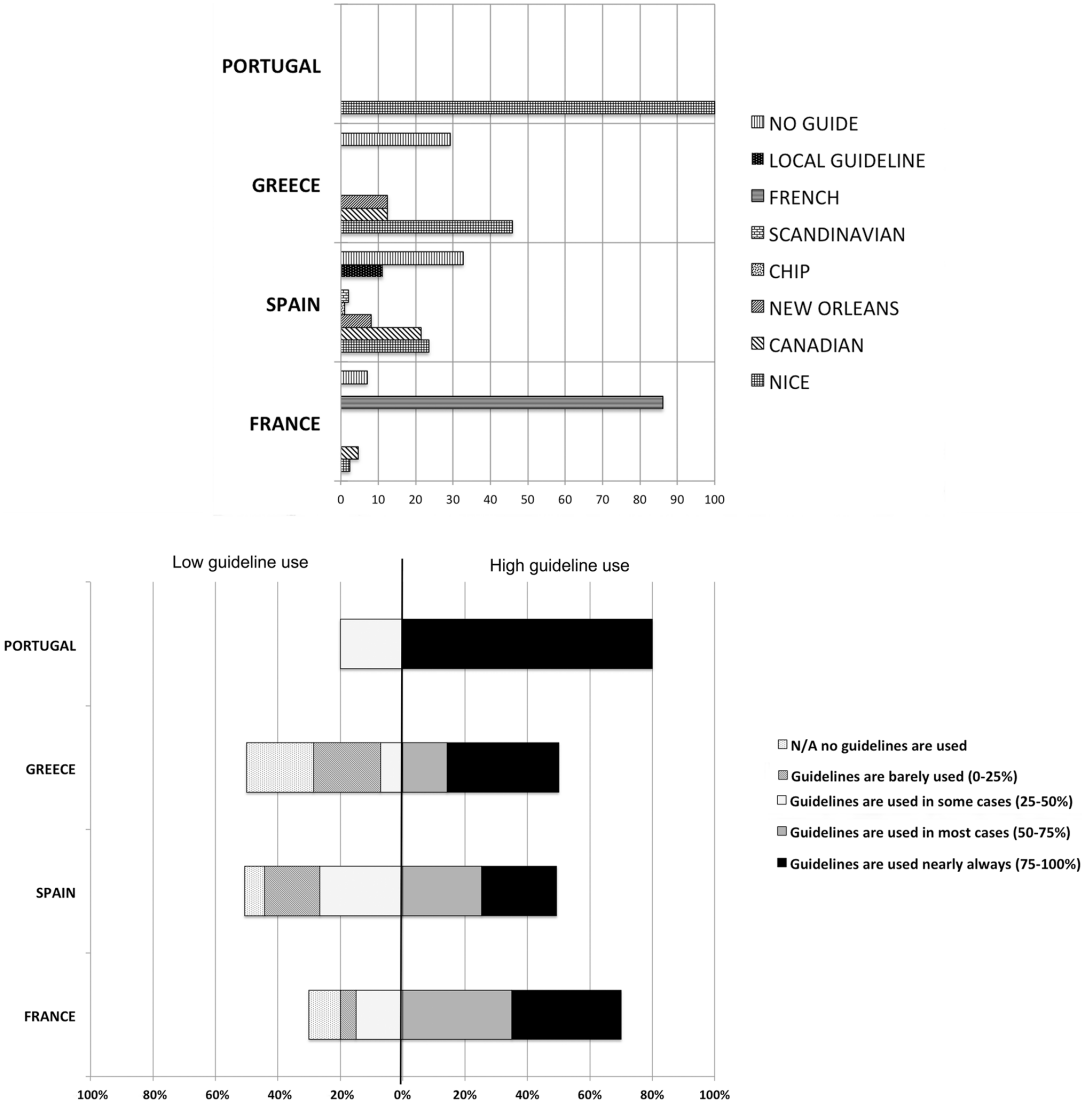
Guideline use and guideline adherence among four European countries surveyed

There is a wide variability in the guidelines declared to be used by the different surveyed centres (Fig. 1). In France and Portugal higher homogeneity was seen where consensual local French guidelines and NICE guidelines seem to be more frequently used. In Greece also NICE guidelines are more frequently used. A wide diversity of guidelines are used in Spain. In Spain as many as 30% of the centres declared that they do not follow any specific guideline. Regarding the estimated guideline compliance, both Portugal and France seem to be the countries that adhere most to guidelines. Greece and Spain did not reach 50% of estimated good guideline compliance (less than 50% of centres estimated guidelines are used in most cases or always, more than 50% adherence to guidelines).

### Factors that prompt CT ordering in mTBI management

**Fig. 2:**
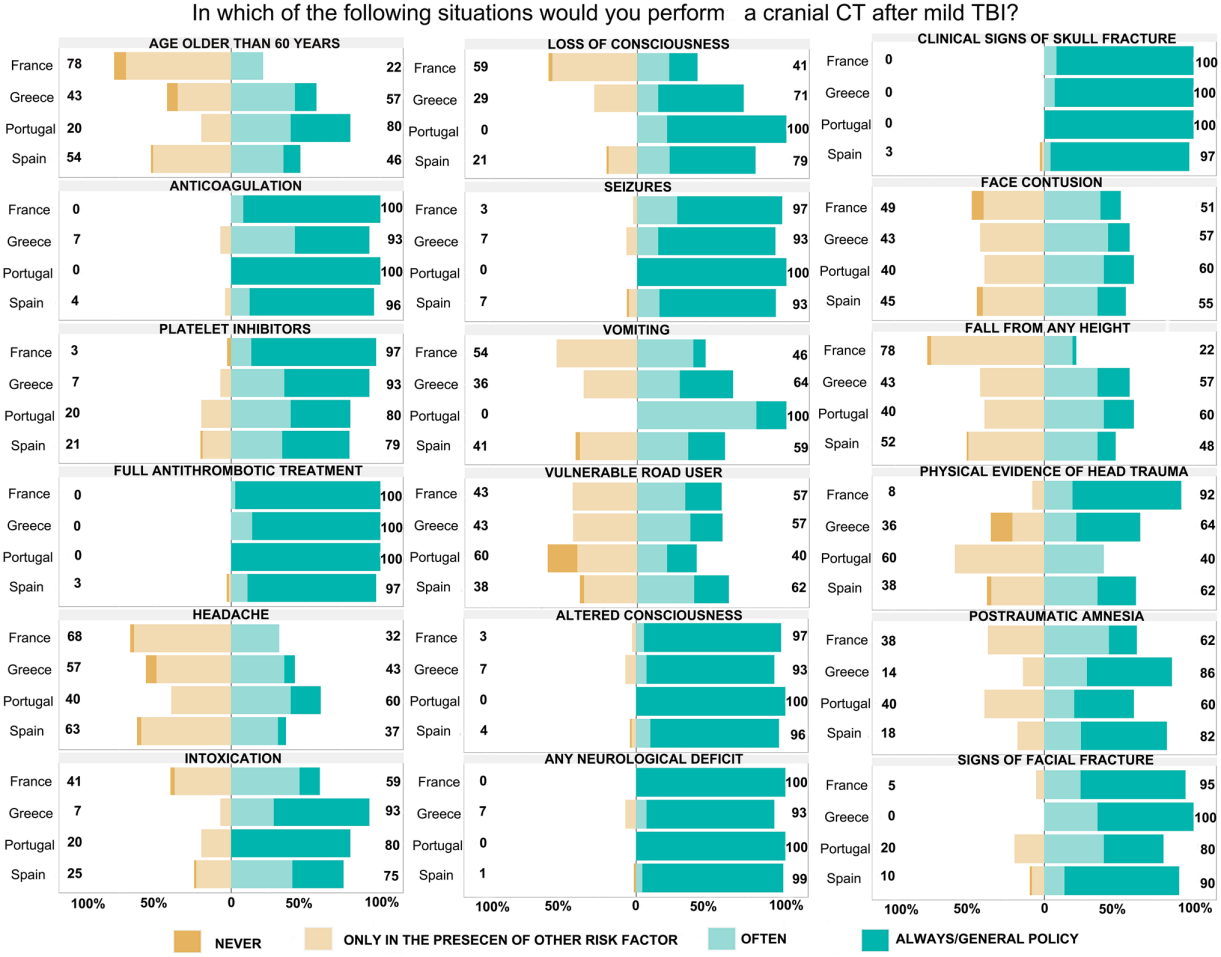
Factors determining the use of cranial CT

When specifically asked about which factors prompt CT ordering in mTBI a number of different areas of agreement and disagreement among physicians working in different countries was noted (Fig. 2). Most physicians would ask for a CT in anticoagulated patients, but also in patients on platelet inhibitors. CT would also be performed if there are seizures, altered consciousness, any neurological deficit, clinical signs of skull fracture or signs of facial fracture. There is variability in the decision to ask for a CT, but not differences among countries, when the patient is older than 60 years of age, report headaches, have episodes of vomiting, in the occasion of a vulnerable road user (pedestrian or cyclist) that was injured by a passing vehicle, if there are facial contusions, or physical evidence of head trauma or face contusions. Though most physicians would perform a CT in case of a patient presenting with intoxication (altered consciousness due to alcohol, drugs or medications) the percentage of physicians that would perform a CT often or always is smaller in France (59% vs over 75% in the rest of the countries). There are significant differences in the indication for CT scan after mild TBI among countries when there is loss of consciousness or posttraumatic amnesia. In both circumstances Spanish doctors would ask more frequently for a CT than the French (*p* < 0.05 bootstrap correction for multiple testing).

### Response to clinical vignettes

**Fig. 3:**
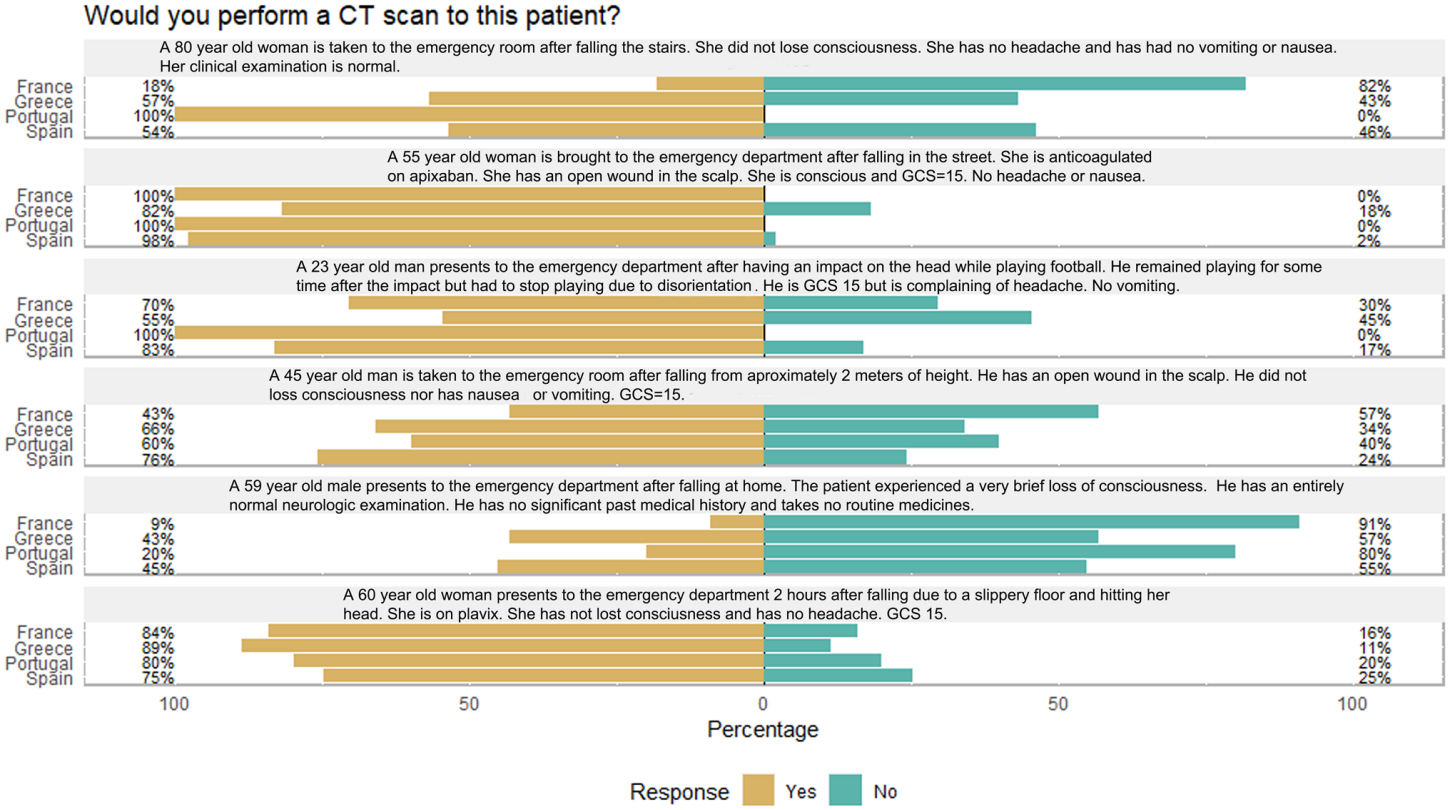
Responses to clinical vignettes among physicians

To better understand physicians’ answers in view of the declared guidelines used, a summary of the factors acknowledged as indication for brain CT by these guidelines and the theoretical response to clinical vignettes following these guidelines is presented in Tables 2 and 3. All physicians’ answers (188) are shown in Fig. 3. There was agreement in most physicians in the different countries for the patient treated with anticoagulant or platelet inhibitors. Though anticoagulant use is a factor in all guidelines for performing a cranial CT, this is not the case with platelet inhibitors only cited by the Scandinavian Guidelines as a factor that combined with advanced age should determine the indication for a cranial CT. Disorientation in sports-related mTBI would prompt the performance of a cranial CT, though this is not really covered by any guideline. Also, falling from more than a person height show a similar result but with wide variability among physicians, possibly reflecting the lack of agreement between guidelines explicitly citing this factor (dangerous mechanism) as indication for cranial CT. Significant differences among countries regarding cases in which age and loss of consciousness are the main reason for performing a CT scan were seen (*p* < 0.05 bootstrap correction for multiple testing) Spanish physicians would perform a CT scan in these patients with a higher frequency and French physicians with lowest frequency than the other countries, in accordance with the wide use of the French guideline which does not include age as a factor for performing cranial CT in these patients..

**Table 2:** Summary of the different factors that prompt the performance of cranial CT after mild TBI in the different management guidelines

	Canadian CT head rule	New Orleans criteria	NICE	French guideline	Scandinavian guideline
Stand alone Indications for CT after mild TBI explicit in guideline					
GCS < 15 at 2 h after injury	✓	✓	✓	✓	S100B*; ≤ 13
Seizures	✓	✓	✓	✓	✓
Anticoagulation	✓	✓	✓	✓	✓
Bleeding disorders	✓	✓	✓	✓	✓
Suspected cranial fracture (skull/depressed/cranial base)	✓	✓	✓		✓
Dangerous mechanism (fall from height, pedestrian)	✓		✓		
Age > 60 years	Age > 65	✓	Age > 65		
Vomiting (episodes)	≥ 2	✓	> 1	> 1	S100B*
Retrograde amnesia	✓	✓	> 30 min	> 30 min	
Headache		✓			
Intoxication		✓		✓	
Loss of consciousness	†	†		†	S100B*
Evidence of trauma above clavicles		✓			
Combined factors that indicate CT					
Age 65 and antiplatelet medication					✓

*S100B: CT should be performed if S100 is not available, or not performed < 6 h, or there is extracranial injury, or S100B performed < 6 h after injury is > 0.10 µg/l

†Canadian CT rule, New Orleans and French Guidelines include loss of consciousness as a factor for defining mild TBI which needs further attention

**Table 3:** Theoretical response to clinical vignettes following different mTBI management guidelines

Clinical vignette	Canadian CT head rule	New Orleans criteria	NICE	French guideline	Scandinavian guideline
A 80-year-old woman is taken to the emergency room after falling from stairs. She did not lose consciousness, she has no headache and has no vomiting or nausea. Her clinical examination is normal	✓	✓	✓		
A 55-year-old woman is brought to the emergency department after falling from standing height in the street. She is anticoagulated on apixaban. She has an open wound in the scalp. She is conscious and GCS = 15. No headache or nausea	✓	✓	✓	✓	✓
A 23-year-old man presented to the emergency department after having an impact on the head while playing football. He remained playing for some time but had to stop playing due to disorientation. He is GCS = 15 but is complaining of headache. No vomiting	Disorientation after TBI and TBI in sports are not covered in the guidelines. There is no clear indication for CT with these guidelines				
A 45-year-old man is taken to the emergency room after falling from approximately 2 m of height. He has an open wound in the scalp. He did not lose consciousness nor has nausea or vomiting. GCS = 15	✓		✓		
A 59-year-old male presented to the emergency department after falling at home. The patient experienced a brief loss of consciousness. He has an entirely normal neurologic examination. He has no significant past medical history and takes no routine medicines					
A 60-year-old woman presented to the emergency department 2 h after falling due to a slippery floor and hitting her head. She is on plavix. She has not loss of consciousness and has no headaches. GCS = 15	No CT indication. Only Scandinavian Guidelines use antiplatelet treatment but in conjunction with age > 65				

✓ Indication for CT scan after mild TBI per guideline criteria

### Estimated usage of CT and S100B in the management of mTBI

**Fig. 4:**
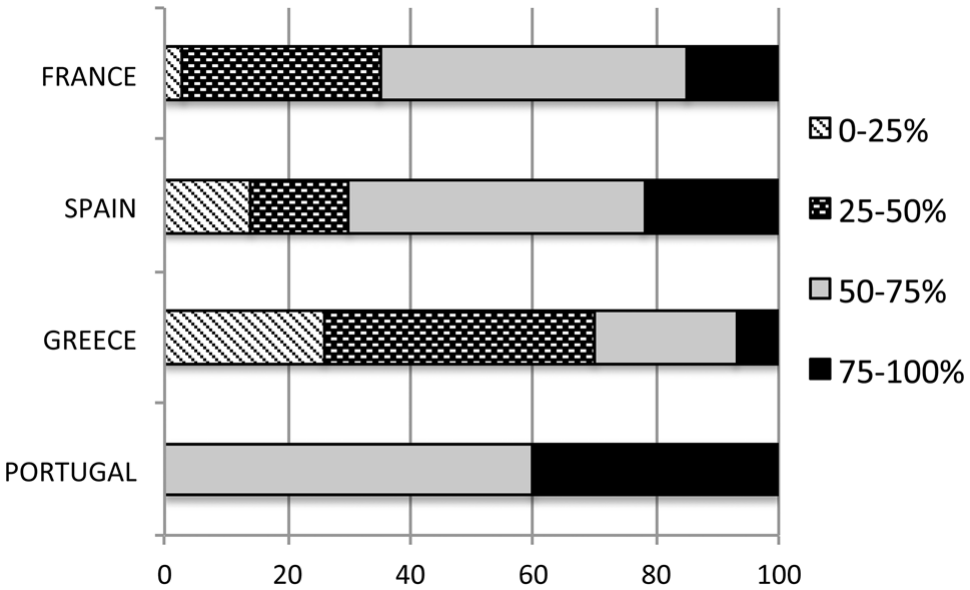
Physician perceived use of CT in mild TBI management

When asked which proportion of mTBI cases would get a CT scan finally at their centres, most physicians answered than in more than 50% of the cases a CT scan would be performed. Therefore, the usage of CT scan for the management of mTBI is very high (Fig. 4). However, when asked if this proportion was appropriate or not, most physicians felt it to be appropriate. Only a large proportion of French doctors (47% of the answers) deemed this proportion to be too high. The use of CT seems to be related to guideline compliance as centres in which no guidelines were used or reported low guideline compliance (less than 25% of cases) used less frequently CT scan in their patients (57% responded to use CT in less than 50% of mTBI cases) than those reporting higher guideline compliance (among centres reporting 50% or more adherence to guidelines; 68% use CT in more than in 50% of mTBI).

The use of S100B in the surveyed countries is anecdotal. Only five centres in France (out of 36, 14%) used routinely S100B for determining the need for cranial CT in mTBI patients. Most of physicians answered that they did not use S100B due to lack of availability in their centres or lack of knowledge about the test. Therefore, despite being in the market for several years, this solution has not been adopted in these countries.

## Discussion

This study shows that management and guideline adherence in mTBI vary a lot in Southern Europe. There is also variability on the speciality responsible for taking care of these patients. No definite consensus on certain conditions that could determine the use of CT in mTBI patients exists, since numerous differences regarding the practical use of CT when physicians are confronted to clinical scenarios were noted. A possible objective means of structuring mTBI management at a biomarker level (i.e., S100B) has not been established in the surveyed countries.

The ED is the first and frequently sole point of medical contact for patients sustaining a mTBI. Though it would seem logical to give an important attention to this health problem, as it is a common cause of consultation to EDs, it seems little attention is paid in many countries. There is a significant local variability regarding which speciality takes care of these patients (emergency medicine, neurosurgery, general surgery, etc.), which could contribute to variability in management and difficulty in offering proper training to physicians. The development of evidenced-based guidelines to manage these patients could contribute in standardizing the management of mTBI and thus decrease variability [5, 7, 8]. However, there is no consensus regarding which guideline to use [11, 18–20]. This factor would not actually compromise management, as the different guidelines present similar criteria to perform CT after mTBI. However, there are developed countries like Spain that declare in a high percentage of cases that centres do not follow any official guideline, and only a minority of centres declare that guidelines are used in most or the majority of their cases.

The available guidelines provide criteria to perform CT based on factors related to patient´s characteristics, like age or the use of anticoagulants, factors related to clinical presentation like trauma mechanisms, presence of seizures, loss of consciousness or signs on examination like altered level of consciousness, GCS < 15 or focal neurological signs [7, 8, 10]. In the current survey, the responses to clinical scenarios made clear that physicians do not agree on age as a sole factor for asking for a CT and physicians do not follow recommendations based exclusively on the loss of consciousness. Physicians seem to believe that the use of antiplatelet agents has a similar effect on terms of presence of intracranial lesions than anticoagulant therapy based on the response to one of the cases.

The findings in this study are in line with previous studies on the management of mTBI in Europe and the US, in which large variations in the assessment and management of mTBI have been detected [14, 20–22]. Moreover areas of controversy previously detected are once again highlighted in the current report, such as age as an indication of cranial CT, loss of consciousness, altered consciousness due to intoxication or the use of platelet inhibitors a factor that is not included in any guideline. It seems that physicians feel safer using CT as screening for these patients, not only for medical reasons but also due to possible malpractice claims, and though most of them feel appropriate to use CT in more than 50% of mTBI cases presenting to their EDs, this could add to CT overuse and excessive exposure to radiation [23].

The results of this survey also point out on the median time spent for mTBI management in the ED, which is 8 h. This can represent a long emergency department waiting time for patients, and substantial health resource use for healthcare systems. Part of this time can be attributed to CT waiting, interpretation and analysis. Though radiological interpretation by emergency physicians is possible, in many centres there is a need for neuroradiological interpretation of CT findings to be able to discharge a patient from ED. With higher selectivity for CT ordering some of this time could potentially be decreased for at least a considerable proportion of patients.

Although extensive research has been performed concluding to recommend S100B as a means of selecting patients for brain CT scan when in doubt or in grey zone areas [13, 24, 25], its use in a wide region of Southern Europe is limited, as has been previously described in another survey including selected level 1 trauma centres [14]. One of the main reasons for not using this test is its lack of availability.

All above findings give even more importance for medical education for physicians taking care of these patients, in terms of achieving a standardized approach regardless of medical speciality or country. A pan-European multidisciplinary consensus guideline could be feasible and would be ideal to standardize management. There are efforts from different organizations to include specific training for physicians managing these patients in the ED, such as the Emergency Neurological Life Support (ENLS) course from the Neurocritical Care Society, or the Global Neuro Diploma in Neurotrauma Care. In both instances, multidisciplinary approaches try to provide physicians in the ED with a set of protocols, checklists and decision points to standardize the management of these patients around the globe. This study also highlights the need for obtaining an unbiased, quick, radiation free an easy to use test to determine the need for CT, observation or admission for these patients [26, 27].

This study has several limitations. Fist, although we tried to include different Hospitals in terms of sizes and available resources to increase generalizability of the results, limited number of Hospitals were reached in Greece and Portugal. However, the results from France and Spain include a large number of centres with wide geographical distribution in the country and including Hospitals of different sizes. Another limitation is the reliability of the answers from physicians regarding guideline use and adherence. The inclusion of clinical vignettes could have enhanced the contextualization of factors prompting CT prescription in these patients, though there is still some limitation concerning the cases involving intoxication, which were not included in the vignettes.

## Conclusions

There are large variations in the assessment of mTBI in the European countries surveyed, regarding guideline use and adherence, and individual factors determining the use of CT as screening tool in these patients. This variation could determine CT overuse. S100B is not used in the majority of European countries. There is a clear need for medical education in Europe to clarify the management of this common clinical problem.

## Supplementary Information

Below is the link to the electronic supplementary material.

Supplementary file1 (PDF 54 KB)

## Data Availability

Data are available on reasonable request. Centre anonymised data are available on reasonable request. Data will be provided for health care policies preparation, or to be part of larger studies.
