# Supplementary material for: Variability in the indication of brain CT scan after mild traumatic brain injury. A transnational survey
Source: Eur J Trauma Emerg Surg. 2022 Feb 18;49(3):1189–98. doi: 10.1007/s00068-022-01902-5 (PMC10229664; doi:10.1007/s00068-022-01902-5)
Supplement: Supplementary file 1 — Supplementary file1 (PDF 54 KB) [file 68_2022_1902_MOESM1_ESM.pdf]

Professional Questionnaire

This survey will allow us to enhance our knowledge on the management of mild TBI in Europe.

Thank you from the BRAINI team!

---

1) What is your speciality?
- ◯ Emergency Department (ED) physician
- ◯ Neurologist
- ◯ Neurosurgeon
- ◯ Trauma surgeon
- ◯ General surgeon
- ◯ Intensive care medicine

---

2) Level of experience
- ◯ Resident
- ◯ Staff

---

3) In which country is your Hospital?

____

---

4) Name of your Hospital

____

---

5) Numer of beds
- ◯ < 300 beds
- ◯ 300-500 beds
- ◯ 500-1000 beds
- ◯ >1000 beds

---

6) Type of Hospital
- ◯ Primary-level hospital(few specialities, limited lab or radiological sevices available)
- ◯ Secondary -level hospital(5-10 specialities)
- ◯ Tertiary-level hospital(highly specialized staff and technical equipment, academic or teaching hospital, national hospital)

---

7) What system of funding supports your Hospital?
- ☐ Public, Government
- ☐ Private

---

8) What is the average number of Emergency Department (ED) visits in your Hospital a day?

____

---

9) What is the average number of mild traumatic brain injury patients visiting the ED in your hospital a day?

____

---

10) In your hospital do you have....
- ☐ Neurosurgeon on call 24/7
- ☐ ICU for trauma patients
- ☐ Department of Neurosurgery

---

11) What speciality is most often responsible for the management(diagnosis, treatment, discharge) of mild Traumatic Brain Injury (mTBI) patients in your hospital in the emergency room(you can sign more than one)
- ☐ Emergency Department (ED) physician
- ☐ Neurologist
- ☐ Neurosurgeon
- ☐ Trauma surgeon
- ☐ General surgeon
- ☐ Intensive care medicine

12) Is a computer tomography technician/specialist available 24/7 to perform a CT scan?

- ◯ No
- ◯ Yes. There is 24/7 in-house availability to perform CT scans.
- ◯ Yes, there is a technician on call and would arrive within 30 minutes to perform CT scans.
- ◯ Yes there is a technician, but it will take longer.

13) Do you have a facility for observation in the ER?

- ◯ No
- ◯ Yes

14) If yes, How many observation beds do you have?

____

15) What is the maximal observation time in this facility?

- ◯ < 6 hours
- ◯ 7-12 hours
- ◯ 13-24 hours
- ◯ Overnight

16) What lowest Glasgow coma scale (GCS) score is considered as mild TBI in your Hospital

- ◯ 15
- ◯ 14
- ◯ 13
- ◯ 12
- ◯ 11
- ◯ 10

17) Are (inter)national or local guidelines used to determine which miltd Traumatic Brain Injury (mTBI) should have an initial head CT in your Emergency Department (ED)?

- ☐ We do not use guidelines
- ☐ NICE
- ☐ Canadian CT head rule
- ☐ New Orleans criteria
- ☐ CHIP rule
- ☐ Scandinavian guidelines for initial management of minimal, mild and moderate head injury
- ☐ Other guides

18) If other guidelines used please state which one:

____

19) How you consider the adherence to the CT guidelines Guidelines at your Emergency Department (ED)?

- ◯ Guidelines are used in almost no cases (0-25%)
- ◯ Guidelines are used in some cases (25-50%)
- ◯ Guidelines are used in most cases (50-75%)
- ◯ Guidelines are used in (almost) all cases (75-100%)
- ◯ N/A: Guidelines are not implemented at our ED.

In which of the following situations would you perform a CT scan in a mild Traumatic Brain Injury patient (TBI) patient?
Select NEVER in factors considered not important in the treatment decision wether someone should get a CT scan.
Select ONLY IN THE PRESENCE OF OTHER RISK FACTORS if the factor is never solely a reason for a CT scan, but it might be a reason in combination with one or more other risk factors.
Select OFTEN/PARTIAL if the risk factor is often seen as a reason for CT scanning in your Hospital.
Select ALWAYS/GENERAL POLICY when the criteria are, in general, a reason for CT scanning in your Hospital (>75% of the paeitnes with this indication)

	NEVER	ONLY IN THE PRESENCE OF OTHER RISK FACTOR	OFTEN/PARTIAL	ALWAYS /GENERAL POLICY
20) Prior loss of consciousness	◯	◯	◯	◯
21) Headache	◯	◯	◯	◯
22) Vomiting	◯	◯	◯	◯
23) Age>60	◯	◯	◯	◯
24) Any anticoagulant therapy (not including anti-platelet therapy)	◯	◯	◯	◯
25) Any antiplatelet therapy (not including anticoagulant therapy)	◯	◯	◯	◯
26) Full anticoagulation(antiplatelet+anti coagulan therapy)	◯	◯	◯	◯
27) Intoxication (alcohol/drugs)	◯	◯	◯	◯
28) Seizure	◯	◯	◯	◯
29) Vulnarable road user (pedestrian or cyclist)	◯	◯	◯	◯
30) Fall from any elevation	◯	◯	◯	◯
31) Post traumatic amnesia	◯	◯	◯	◯
32) Altered consciousness	◯	◯	◯	◯
33) Any neurological deficit	◯	◯	◯	◯
34) Clinical signs of fractures skull	◯	◯	◯	◯
35) Physical evidence of trauma to head/skull	◯	◯	◯	◯
36) Signs of facial fracture	◯	◯	◯	◯
37) Contusions of face	◯	◯	◯	◯

38) What percentage of all mild Traumatic Brain Injury(TBI) patients attending your Emergency Department (ED) do get a CT scan?

- ◯ 0-25%
- ◯ 25-50%
- ◯ 50-75%
- ◯ 75-100%

39) Do you feel this proportion of CTs is.....

- ◯ Too high
- ◯ Appropriate
- ◯ Too low
- ◯ Don´t know

40) Is S100B routinely determined as a prognostic biomarker or to guide indication for CT?

◯ No
◯ Yes

41) Why do you not use S100

☐ Availability
☐ Price
☐ Lack of confidence in performance
☐ Not aware of the test

42) What is the average time for the diagnostic workout of a mild TBI patient in your Hospital before discharge?
Please state the amount in hours

____

43) In the event of having a new diagnostic tool for mild TBI what would be your highest priority for it

☐ High negative predictive value for deterioration ☐ High negative predictive value for detecting brain lesions
☐ High ability for prognostication
☐ Speed and comfort for the patient

CLINICAL VIGNETTES: PLEASE READ THE VIGNETTE AND ANSWER IF YOU WOULD ORDER A CT SCAN OR NOT.
A 59 year old male presents to the emergency department after falling at home. The patient tripped on a shoe in his living room and fell, striking his head on a wooden floor. The event happened 1 hour ago. The patient experienced a very brief loss of consciousness. The patient has no headache and has had no nausea or vomiting. He has an entirely normal neurologic examination. He has no significant past medical history and takes no routine medicines.

44) Would you perform a head CT scan on this patient?

◯ Yes
◯ No

A 80 year old woman is taken to the emergency room after falling the stairs. She did not lose consciousness. She has no headache and has had no vomiting or nausea. Her clinical examination is normal.

45) Would you perform a head CT scan on this patient?

◯ Yes
◯ No

A 23 year old man presents to the emergency department after having an impact on the head while playing football. He remained playing for some time after the impact but had to stop playing due to de-orientation. He is GCS 15 but is complaining of headache. No vomiting.

46) Would you perform a head CT scan on this patient?

◯ Yes
◯ No

A 60 year old woman presents to the emergency department 2 hours after falling due to a slippery floor and hitting her head. She is on plavix. She has not lost consciusness and has no headache. GCS 15.

47) Would you perform a head CT scan on this patient?
◯ Yes
◯ No

A 45 year old man is taken to the emergency room after falling from aproximately 2 meters of height. He has an open wound in the scalp. He did not loss consciousness nor has nausea nor vomiting. GCS=15.

48) Would you perform a head CT scan on this patient?
◯ Yes
◯ No

A 55 year old woman is brought to the emergency department after falling in the street. She is anticoagulated on apixaban. She has an open wound in the scalp. She is conscious and GCS=15. No headache or nausea.

49) Would you perform a head CT scan on this patient?
◯ Yes
◯ No
